# High Precision Vibration-Type Densitometers Based on Pulsed Excitation Measurements †

**DOI:** 10.3390/s19071627

**Published:** 2019-04-05

**Authors:** Andreas Rechberger, Robert Amsüss, Stefan Rossegger, Robert Breidler, Gerald Steiner

**Affiliations:** Anton Paar GmbH, 8054 Graz, Austria; robert.amsuess@anton-paar.com (R.A.); stefan.rossegger@anton-paar.com (S.R.); robert.breidler@anton-paar.com (R.B.); gerald.steiner@anton-paar.com (G.S.)

**Keywords:** flexural resonator, pulsed excitation, repeated fade-out, density measurement, fluids, viscosity correction

## Abstract

Glass flexural resonators have established themselves as one of the de-facto standard methods for measuring the density of liquids in a laboratory environment. The core of this sensor is a U-Tube measuring cell whose oscillator’s resonance frequency changes with the mass of the liquid within the tube. This relationship can be used to derive the density of liquids in a fast and reliable way. In order to achieve the highest accuracy for the density measurement multiple physical effects (e.g., damping due to viscosity effects) need to be taken into account. For a reliable correction, additional measurements are required. The pulsed excitation method is able to produce these additional parameters along with a superior measurement performance compared to previous techniques.

## 1. Introduction

Densitometers based on U-tube sensors [1] are used in a multitude of applications. These include but are not limited to monitoring the seawater properties for means of climate monitoring [2], flow metering and fluid custody transfer [3], as well as for accurate modelling of industrial processes [4]. For efficient design of chemical processes, and calculations for flow conditions and hydrostatic stress, liquid density data is required. While many methods for measuring the fluid density are known, they are often time consuming. Density determination based on the hydrostatic principle is known to achieve the highest accuracy for measuring the density of liquids. Apart from long measurement time, it requires a large amount of the liquid to be measured. A vibrating tube density meter is based on a bent tube that is filled with the liquid to be measured. The tube is brought to resonant oscillation by exerting a suitable force on the tube. The liquid under test changes the mass of the system and hence the resonance frequency of the glass tube. Since the volume of the glass tube is kept constant, there is a direct relationship between the measured resonance frequency and the density of the liquid. Especially by employing digital measurement techniques the drawbacks of pycnometers, glass hydrometers and hydrostatic weighing can be mitigated. The benefits are lower sample volume, reduced handling impact for better reproducibility, and improved compensation of temperature related effects.

As the understanding of this principle has matured over the last decades, the ambition to constantly achieve more accurate results has persistently demanded improvements to both the measurement techniques as well as the physical models.

The calculation of the density is based on the fundamental Equation (1) that relates the period of an oscillation mode (τ) with the density of the filled fluid. Prior to a density measurement, the device constants *A* and *B* have to be determined with fluids of known density. Since this simple relationship does not account for, as an example, the effects of the viscosity of the fluid on the resonance frequency, corrections to the model are necessary [5]. Moreover by acquiring data from additional modes of vibration the accuracy of density results can be improved.

(1) $$\rho =A\tau^{2}+B$$

One major improvement in the last decades was to add another glass oscillator to the measurement scheme that is deliberately not filled with the liquid under test. This additional oscillator acts as a reference and therefore drastically improves the accuracy. To sum up the total number of oscillation parameters that have to be determined in a single density measurement has increased over time.

The state of the art in current densitometers is the determination of up to four parameters of the resonating U-tube. Those parameters are the fundamental frequency, the first harmonic frequency, and the frequency of the reference oscillator the damping ratio for one of those modes.

Within this work, an enhanced approach for the characteristics of a resonator is presented. It allows a faster and more accurate determination of the resonance parameters (frequency and damping ratio), as well as offers the possibility to measure up to four parameters per mode simultaneously. Resonators designed for high end devices commonly use three modes, which results in 12 parameters available for density and viscosity determination.

## 2. Signal Model

**Figure 1:**
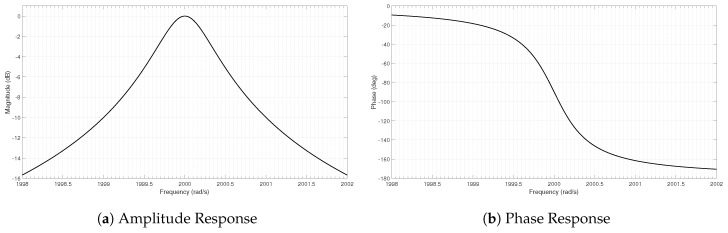
Amplitude and Phase response of the theoretical model close to the resonance frequency.

The signal model of a single oscillation mode exhibits a Lorentzian band pass behaviour as sketched in Figure 1.

In order to determine the desired parameters (resonance frequency $\omega_{0}$ and the damping ratio ζ or attenuation rate α (6)), at least two points of the curve need to be known. For practical reasons one of those points is the resonance frequency $\omega_{0}$. With a feedback-based actuation system, the excitation signal can be arbitrarily placed on the desired phase condition. In order to extract the damping ratio of a system in addition to exciting the resonator at its resonance frequency (phase condition ΔΦ=−π/2), it is required to superpose additional frequency components [6]. Suitably this added frequency components are chosen according to (2), with *B* being the bandwidth of the resonator and (4) denoting the required phase condition for these two frequencies. By utilizing the relation given in (5) the quality factor *Q* can be computed from the frequency difference.

(2) $$f_{1,2}=f_{0}\pm \frac{B}{2}$$

(3) $$B=\Delta f=|f_{2}-f_{1}|$$

(4) $$\Delta \Phi \left(f_{1,2}\right)=-\frac{\pi }{2}\pm \frac{\pi }{4}$$

(5) $$Q=\frac{f_{0}}{\Delta f}=\frac{1}{2\zeta }$$

(6) $$\zeta =\frac{\alpha }{\omega_{0}}$$

For a typical U-tube suited for densitometers, the system exhibits very high quality factors. Exemplarily the system depicted in Figure 1 represents a resonance frequency of 2000 rad/s, and a quality factor of 1000. In terms of the desired measurement range this represents an average case.

When exciting the resonator at frequencies $f_{0}$, $f_{1}$ and $f_{2}$ simultaneously, these frequencies can not be directly measured as they are very close to each other. Separating these frequencies by means of band pass filters [6] in such a case is a very challenging task. The quite small bandwidth ($|f_{2}-f_{1}|<0.5\mathrm{rad}/s$ at its minimum) of the resonator demands an comparable bandwidth of the channel selection filters. Further their band edge needs to be very sharp in order to offer sufficient selectivity in between the side band signals and the carrier. This demands filters with high order. With such an approach problems such as the computational effort and potential instabilities cannot be neglected. Even when ignoring design issues, such a system would intrinsically be very slow caused by the necessity of very narrow band filters.

## 3. Continuous Excitation

A common solution is to measure the bandwidth of the system by indirect means, such as modulating the excitation signal. With a simple amplitude modulated drive signal, a symmetric side band signal can be generated. Demodulating the response leads to the resonator’s bandwidth [7].

So far both approaches, direct and indirect measurement, are based on the principle of generating a suitable excitation signal, and measuring the system’s response. In order to reach the highest accuracy, some systematic problems of this approach have to be taken into account.

Whenever a control loop is used to generate the drive signal, the accuracy of the measured resonance frequency depends on the absolute error of the phase measurement. While suitably designed controllers usually do not experience any steady state error, the same does not hold true for the sensing electronics. For a simple model of a digitally controlled measurement engine, the excitation signal is composed by digital synthesis and applied to the resonator by means of digital to analogue conversion and amplification. In addition, the sensing signal requires an analogue preprocessing prior to analogue to digital conversion. The frequency response of the analogue front end can only be partially compensated, which leads to an incorrectly measured frequency $f_{0}$. For the measurement of the resonant frequency alone this error is of no relevance in the majority of applications. It is either too small to have an appreciable impact on the measurement result or can be eliminated by means of suitable adjustments.

However, when applying an amplitude modulated drive signal (7) whose carrier does not perfectly match the resonance frequency of the U-tube (${\hat{\omega }}_{0}\neq \omega_{0}$), the upper and lower side band no longer experience an identical attenuation and phase shift.

(7) $$\begin{array}{cc} x(t) & =\sin\left({\hat{\omega }}_{0}t\right)\left(1+m\sin\left(\omega_{m}t\right)\right)\\ & =\sin\left({\hat{\omega }}_{0}t\right)+m\sin\left(\left({\hat{\omega }}_{0}\pm \omega_{m}\right)t\right) \end{array}$$

(8) $$\begin{array}{cc} y(t) & =|G\left({\hat{\omega }}_{0}\right)|\sin\left({\hat{\omega }}_{0}t+\arg G\left({\hat{\omega }}_{0}\right)\right)\\ & +|G\left({\hat{\omega }}_{0}+\omega_{m}\right)|m\sin\left(\left({\hat{\omega }}_{0}+\omega_{m}\right)t+\arg G\left({\hat{\omega }}_{0}+\omega_{m}\right)\right)\\ & +|G\left({\hat{\omega }}_{0}-\omega_{m}\right)|m\sin\left(\left({\hat{\omega }}_{0}-\omega_{m}\right)t+\arg G\left({\hat{\omega }}_{0}-\omega_{m}\right)\right) \end{array}$$

G(ω) in (8) denotes the complex system response (attenuation and phase shift). With ${\hat{\omega }}_{0}=\omega_{0}+\omega_{error}$ no longer being the exact resonance frequency, $G({\hat{\omega }}_{0}\pm \omega_{m})$ does not resemble a pair of complex conjugates any more. As such the upper side band is attenuated and phase shifted by slightly different values than the lower side band. This causes impairments to the sensing signal y(t).

**Figure 2:**
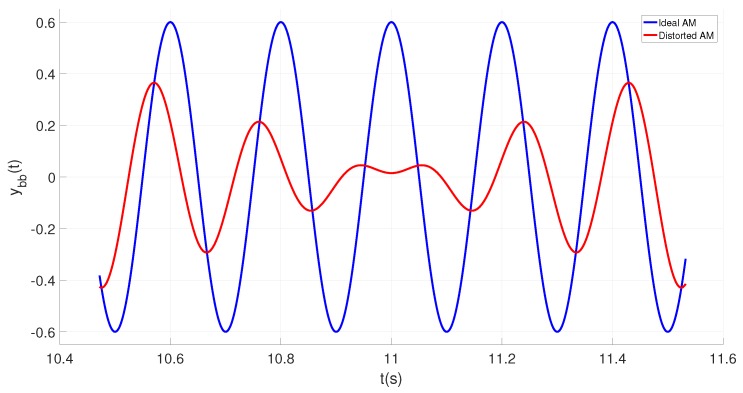
Baseband signal of AM modulation.

Figure 2 depicts the baseband signal of an ideal as well as a distorted amplitude modulated signal. It is a figure of merit for the oscillator quality factor (bandwidth). As soon as the side band impairments are in place, the baseband signal shows a periodic distortion (Figure 2, red line) which will introduce an error to the quality factor measurement. When comparing the ideal (blue line) with the distorted curve (red line) it is apparent that phase as well as amplitude of the distorted base band signal experience a periodic error. During the time frame in which the amplitude approaches zero (e.g., t≈ 11 s), an instantaneous phase detection becomes impossible.

**Figure 3:**
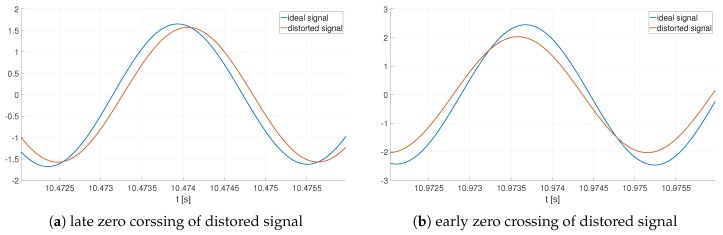
Ideal and distorted sensing signal y(t) with $0.5^{\circ }$ phase and 5% amplitude mismatch.

These impairments do not only influence the quality factor measurement (based on the demodulated side band signal (envelope)), but can also influence the resonance frequency measurement. Figure 3 demonstrates the distorted signal according to (8) in two selected time frames. Alternatively, Figure 3a demonstrates that when taking the zero crossing as reference the ideal signal is ahead of the distorted one (t≈ 10.4732 s). Further, the second time frame ( Figure 3b) depicts the contrary. The ideal signal lags behind the distorted one ((t≈ 10.9727 s).

When using a fast frequency estimation algorithm (such as zero crossing detection or short time Fourier analysis), these fluctuations cause the stability (standard deviation σ) of the measurement to deteriorate. Due to the low frequency of the error pattern, mitigating these effects by means of averaging is a time consuming process.

## 4. Pulsed Excitation

The Pulsed Excitation principle mitigates this problem by withdrawing the concept of measuring the systems response to an externally applied excitation signal. Rather than comparing the drive and sensing signal in a closed loop system, the measurement takes place in two distinct phases.

1. Excitation
  In this phase no measurement is done, the oscillation is built up.
2. Fade-out
  In this phase the deflection of the U-tube is measured.

Even if the U-tube does not oscillate at its exact resonance frequency at the end of the excitation phase, it will approach $\omega_{0}$ very rapidly once the external stimulus has disappeared.

By observing the damped undisturbed free oscillation of the resonator, the quality factor as well as the damped resonant frequency can be determined by direct measurement. With the sensing signal as given in (9) the quality factor directly relates to the attenuation rate α as shown in (11). With knowledge of the quality factor, the undamped resonance frequency can be calculated from the damped resonance frequency (10). Either the damped resonance frequency $\omega_{d}$, or the un-damped resonance frequency $\omega_{0}$, can be worked into the viscosity correction procedure of the sensor.

(9) $$y\left(t\right)=A_{0}e^{-\alpha t}\sin\left(\omega_{d}t+\varphi_{0}\right)+w\left(t\right)$$

(10) $$\omega_{d}=\sqrt{\omega_{0}-\zeta^{2}}$$

(11) $$Q=\frac{\omega_{0}}{2\alpha }$$

**Figure 4:**
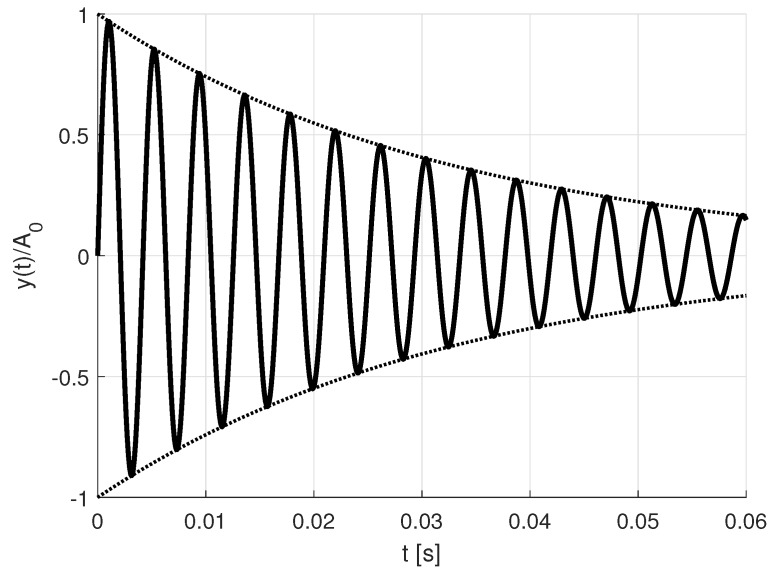
Single damped oscillation.

Figure 4 sketches the output signal of the resonator after disabling the drive signal, omitting the noise w(t) for reasons of better visualization. The superposed decay signal (12) will be present at the sensing circuitry. It represents the combination of all oscillation modes which have been excited.

(12) $$\begin{array}{c} y\left(t\right)=\sum_{i}A_{i}\sin\left(\omega_{i}t+\varphi_{i}\right)+w\left(t\right) \end{array}$$

**Figure 5:**
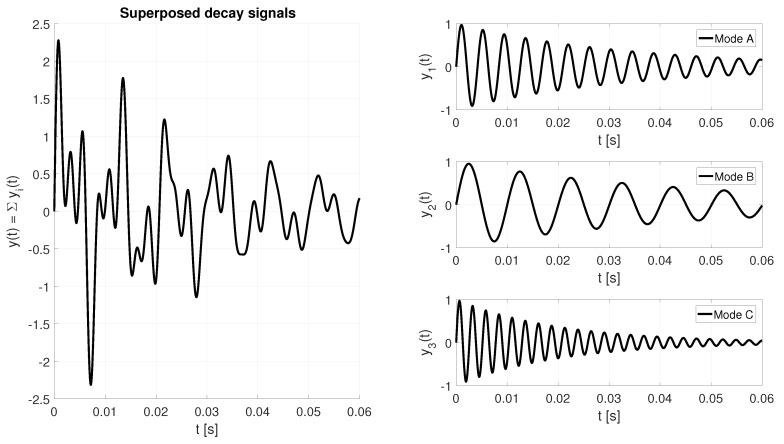
Superposed damped oscillations.

In Figure 5 all three modes are shown with a normalized amplitude ($A_{0}=1$). In practice each mode will experience a different attenuation. Therefore the values of $A_{i}$ are usually adjusted to the specific attenuation of the oscillator arrangement. As with any physical system the excitation signal (13) applied to the resonator has finite energy, and must satisfy (14), where C is a constant expressing the finite output power of the excitation circuitry.

(13) $$x\left(t\right)=\sum_{i}A_{i}\sin\left(\omega_{i}t+\varphi_{i}\right)$$

(14) $$\sum A_{i}\leq C$$

As in this mode of operation there is no feedback required from the sensing signal to the output, the extraction of the attenuation rate and the frequency measurement is an optimization problem of fitting the sensing data y(t) to the model given in (16) rather than a controller property. The determination of $\omega_{d}$ and *Q* can be done by minimizing (17) in the least square sense.

(15) $$P=\left[\begin{array}{cccc} A_{0} & \alpha & \omega_{d} & \phi_{0} \end{array}\right]$$

(16) $$m\left(t,P\right)=P_{0}e^{-P_{1}t}\sin\left(P_{2}t+P_{3}\right)$$

(17) $$S=\sum {\left(x\left(t\right)-m\left(t,P\right)\right)}^{2}$$

With suitable excitation logic, the pulsed excitation principle can be applied to multiple modes in parallel. Modern densitometers usually use the resonators fundamental eigenmode combined with the first harmonic. The specific modes are chosen with respect to their sensitivity to the desired density and viscosity range. For complex resonator arrangements more than two modes could be utilized. Provided that the distinct modes have sufficient frequency spacing, the separation can easily be done by band pass filters. Due to the nature of the measurement principle the amplitude and phase response of these individual filters has minimal impact to the measurement. In particular, there is no requirement on the filters for the individual resonator modes to have matching filter characteristics, such as group delay. Each channel can be served by a band pass filter individually matched to the properties (e.g., bandwidth) of its mode. This is caused by the fact that in difference to closed loop systems, which are in need to perform a phase comparison of the drive signal and the measured response after mode separation, the optimization problem operates in the sensing signal only. For the signal model these multiple oscillation modes simply reflect a superposing of the various damped oscillations, as sketched in Figure 5. It depicts the sensing signal with three decaying oscillations on the left side. As the three modes have significantly different resonance frequencies the sensing signal can be easily post processed by band bass filters. This allows to isolate the various oscillation modes which is shown on the right side of Figure 5.

## 5. Experimental Setup

The pulsed excitation method was successfully implemented in the latest generation of densitometers [8,9,10]. For the measurements referenced in this paper the raw period and Q-Factor data have been extracted from a prototype of the Anton Paar DMA 5001 density device. All measurements are performed with the U-tube kept at 20 ${}^{\circ }C$ constant cell temperature, within a laboratory environment at room temperature between 24 and 25 ${}^{\circ }C$. In order reduce the impact of temperature equilibration each sample was filled into the measurement cell 25 min prior to extracting the measurement data. The fluids and media used are shown in Table 1.

## 6. Results

**Figure 6:**
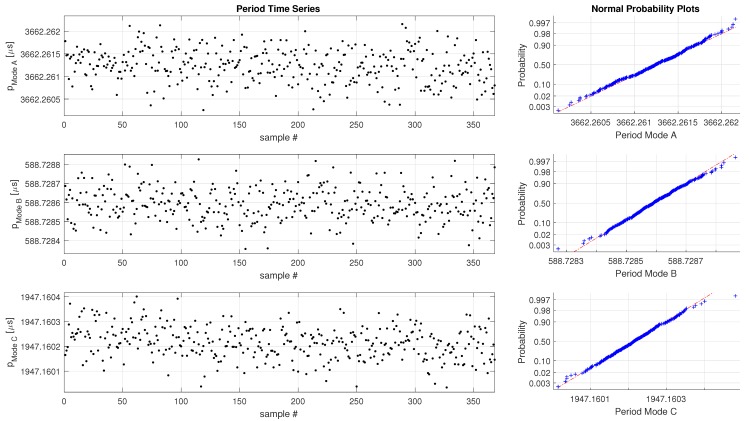
Period data, simultaneous measurement of three modes, U-tube filled with water.

**Figure 7:**
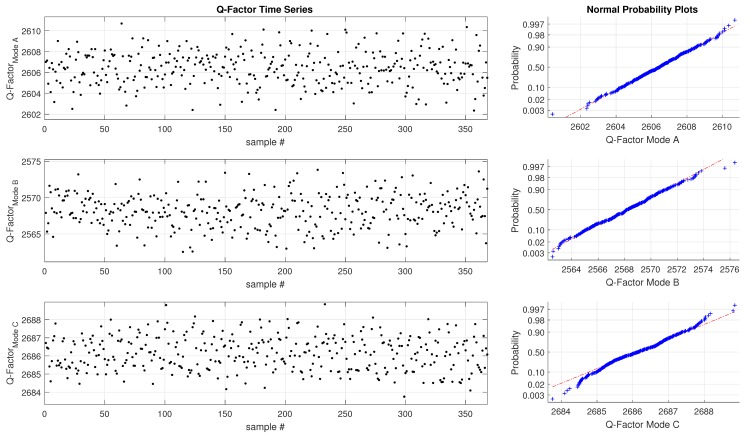
Q-factor data, simultaneous measurement of three modes, U-tube filled with water.

For the water measurement the frequency and quality factor of three simultaneously operated oscillation modes (mode A, B and C) are depicted in s Figure 6 and Figure 7. The time series is depicted on the left, and the corresponding statistical representation on the right side. It can be seen that the raw data from the sensors follows a normal distribution for the period as well as the Q-Factor. In the absence of filling errors, and temperature changes the Pulsed Excitation Method does exhibit only Gaussian noise.

According to (15) there are four parameters for each mode determined during a single excitation-decay sequence. The standard deviation σ of the measurements are given in Table 2. Only six (frequency and Q factor for each mode) out of the 12 possible measurements are shown, the details are shown. The experimental measurements have revealed that even liquids with a large viscosity value can be measured with a reasonably stable with a standard deviation below $0.5\times 10{-}^{9}$ s.

## 7. Conclusions

Using the additional parameters, provided by the Pulsed Excitation Method applied to all three modes simultaneously, allows more accurate measurement of the density. In particular, the effect of viscosity [5] on the period and the density measurement can be corrected. Also features such as detecting inappropriate filling or damaged sensors can be deduced from the combined frequency and damping ratio measurements. For liquids that show a significant sensitivity on the viscosity effect with respect to the observed density, a viscosity correction improved by a factor of two could be achieved when compared to previous approaches [7].

## Figures and Tables

**Table 1:** Media reference data.

Medium	Nominal Density [$\frac{g}{{\mathrm{cm}}^{3}}$]	Nominal Viscosity [mPas]
Reference Oil 1	0.86681	282.1
Reference Oil 2	0.84676	1186
Bromobenzene	1.49488	1.212
Water	0.99820	1.00

**Table 2:** Measurement data for different media.

Medium	Mode	Period [μs]	$\sigma_{\mathrm{period}}$ [ns]	Q-Factor	$\sigma_{Q}$
Water	Mode A	3662.2612	0.37	2606.4	1.6
	Mode B	588.7286	0.09	2568.2	2.4
	Mode C	1947.1602	0.07	2686.1	0.9
Reference Oil 1	Mode A	3541.2762	0.46	2332.5	1.4
	Mode B	569.5514	0.57	933.0	1.7
	Mode C	1947.1601	0.07	2686.3	0.9
Reference Oil 2	Mode A	3522.5131	0.39	2653.6	1.6
	Mode B	566.7755	0.34	1085.8	1.6
	Mode C	1947.1589	0.09	2685.6	0.9
Bromobenzene	Mode A	4088.8993	0.48	2623.2	1.8
	Mode B	657.1678	0.10	2554.9	2.5
	Mode C	1947.1582	0.07	2686.3	0.9
